# Regulatory B Cells Are Decreased and Functionally Impaired in Myasthenia Gravis Patients

**DOI:** 10.3389/fneur.2022.808322

**Published:** 2022-02-28

**Authors:** Ye Lin, Ting Chang, Jiaji Lin, Chenjing Sun, Chao Wei, Jiao Zhao, Rui Liu, Kun Yang, Zhuyi Li

**Affiliations:** ^1^Department of Neurology, Tangdu Hospital, Air Force Medical University, Xi'an, China; ^2^Department of Neurology, Chinese PLA General Hospital, Beijing, China; ^3^Department of Radiology, Chinese PLA General Hospital, Beijing, China; ^4^Department of Rehabilitation, Tangdu Hospital, Air Force Medical University, Xi'an, China; ^5^Department of Immunology, Basic Medicine School, Air Force Medical University, Xi'an, China

**Keywords:** myasthenia gravis, regulatory B cell, IL-10, STAT3, anti-acetylcholine receptor antibody, autoimmune disease

## Abstract

Myasthenia gravis (MG) is an autoimmune disease mediated by B cells secreting autoantibodies. Regulatory B (Breg) cells confirmed to have an immunosuppressive function play an important role in many autoimmune diseases. However, what about the changes in Breg cells in the thymus and peripheral blood of MG patients? The changes in the proportion of Breg cells in the peripheral blood of 41 MG patients without any drug treatment and 30 healthy controls were detected by flow cytometry. We found that the proportions of CD19^+^ IL-10^+^ cells and CD19^+^CD24^hi^CD38^hi^ cell subsets in MG patients were significantly lower than those in healthy controls. Then, we detected the proportion of CD19^+^ IL-10^+^ cells in thymus tissues of 10 healthy children, 4 healthy adults, and 12 MG patients by flow cytometry. However, the percentage of CD19^+^ IL-10^+^ cells was highest in healthy children (~8%), followed by healthy adults (~3%), and was lowest in MG patients (~0.5%). CD19^+^CD24^hi^CD38^hi^ B cells exerted immunosuppressive functions in healthy people but were refractory in MG patients. Moreover, p-STAT3 downstream of CD40 may be impaired in CD24^hi^CD38^hi^ B cells from the peripheral blood of MG patients.

## Introduction

Myasthenia gravis (MG) is an antibody-mediated autoimmune disease. Autoantibody deposition in the neuromuscular junction (NMJ) resulted in the depletion of acetylcholine (ACh) at the NMJ and fluctuating weakness in skeletal muscle groups. Most MG patients have anti-AChR antibodies, and antibodies against MuSK (muscle-specific receptor tyrosine kinase) and LRP4 (low-density lipoprotein receptor-related protein 4) have been found to be involved in the pathogenesis of MG. Approximately 15% of patients have ocular MG (OMG), whereas 85% have more generalized MG (GMG), including non-ocular muscle weakness. Patients can be divided into early-onset (EOMG) and late-onset MG (LOMG) according to the onset age of 50 years (1, 2).

The mechanism of MG is complex and involves the impairment of central immunity (thymus) and peripheral self-tolerance mechanisms. The pathophysiology of AChR-MG is closely related to the abnormal thymic pathology, such as thymic hyperplasia or the presence of thymoma (3). In addition, thymectomy or thymomectomy is recommended in patients with generalized AChR-MG (4). Multiple regulatory pathways that prevent lymphocytic hyperactivation and restrain existing inflammatory signals are pivotal in immune homeostasis. In addition to the well-established contribution of Tregs to the maintenance of immune tolerance, a population of B cells, known as regulatory B (Breg) cells, are immunosuppressive cells that support immunological tolerance (5). The existence of B cells with the capacity to suppress immunology was initially reported in the mid-1970s in studies performed in guinea pigs (6). Breg cells play negative immuneregulatory roles in the development of many autoimmune diseases, such as experimental autoimmune encephalitis (EAE) (7), systemic lupus erythematous (SLE) (8), rheumatoid arthritis (RA) (9), and type 1 diabetes (T1D) (10).

Unlike Treg cells, Breg cells lack specific surface markers. Human Breg cells are still defined by their function of secreting IL-10 (known as B10 cells). In the exploration of the phenotype of Breg cells, CD19^+^CD24^hi^CD38^hi^ B cells were most demonstrated in humans, belonging to immature B cells, and were confirmed to be rich in IL-10-producing cells with regulatory capacity in healthy people. The IL-10-producing B cell subset inhibits the progression of Th2-driven disease by downregulating the inflammatory cascade associated with IL-1 and signal transducer and activator of transcription (STAT)-3 activation (11). CD40 signaling is important for the generation and function of Breg cells in many autoimmune diseases (12).

However, the changes in Breg cells vary among different autoimmune diseases. Some studies have reported that the number of Breg cells in the peripheral blood of patients with MG is decreased (13, 14). To date, there is still no related study on the changes in Breg cells in the thymus of MG patients, so it is unknown whether the function of Breg cells is normal in these patients.

## Methods

### Patients and Controls

Forty-one MG patients were enrolled at The Second Affiliated Hospital of Air-Force Military Medical University from September 2014 to May 2016. The patients included 17 men and 24 women. The age distribution of the patients ranged from 3 to 82 years, with an average age of 41.8 ± 17.2 years. All patients (Table 1) met the MG diagnostic criteria for clinical symptoms, were neostigmine test positive, and were electrophysiologically confirmed. The exclusion criteria were as follows: (i) infection within 1 month; (ii) the presence of other autoimmune diseases (such as multiple sclerosis, rheumatoid arthritis, diabetes, systemic lupus erythematosus, hyperthyroidism, etc.); and (iii) treatment with hormones, immunosuppressants (cyclophosphamide, azathioprine, etc.), intravenous immune globulin, or plasma exchange. Characteristics of the study participants were summarized in Table 2. A total of 30 healthy subjects were recruited as the controls and were matched with the MG patients in terms of age, gender, and demography. All of peripheral blood samples from MG patients were taken before thymic surgery. According to the principles outlined in the Declaration of Helsinki and the ethics committee-approved protocols, all subjects provided written and signed informed consent prior to enrollment. Blood sample collections from human subjects for detailed immunological assessments were approved by the Ethics Committee of Air-Force Military Medical University. All patients and healthy controls provided written informed consent to participate.

**Table 1:** Demographics and clinical characteristics of all patients.

			**Anti-AChR**		**Duration**				**Previous**
**No**.	**Age (years)**	**Gender**	**Ab (nmol/L)**	**Onset**	**(months)**	**MGFA class**	**QMGS**	**Thymus status**	**therapies**
1	64	M	−0.26	LO	18	I	3	None	PB
2	56	M	2.05	LO	4	I	3	None	None
3	25	F	0.7	EO	240	I	3	None	None
4	47	F	6.34	EO	2	IIB	11	Thymoma	None
5	36	M	4.96	EO	4	IIB	7	N.A.	None
6	46	F	0.01	EO	12	IIB	4	Thymic hyperplasia	PB
7	29	M	−0.13	EO	84	IIB	4	Thymic hyperplasia	PB
8	82	M	0.62	LO	36	IIA	3	None	PB
9	34	F	4.22	EO	60	I	8	None	PB
10	28	M	3.84	EO	2	IIB	3	None	None
11	3.7	F	0.1	EO	1	I	5	N.A.	None
12	55	F	4.22	LO	24	IIA	6	N.A.	PB
13	38	F	0.05	EO	4	I	7	None	None
14	57	M	3.91	LO	6	IIA	3	None	None
15	48	M	2.63	EO	24	I	3	Thymic hyperplasia	PB
16	44	F	3.01	EO	2	I	3	Thymic hyperplasia	PB
17	50	M	1.2	LO	9	I	3	None	None
18	41	M	7.56	EO	1	IIB	3	None	None
19	30	F	0.8	EO	24	I	3	None	PB
20	54	F	−0.1	LO	3	I	12	None	None
21	34	M	2.46	EO	3	IIIA	3	Thymoma	None
22	41	F	2.92	EO	14	I	3	None	PB
23	38	M	3.95	EO	2.5	I	6	None	PB
24	54	F	3.84	LO	36	IV	6	Thymoma	PB
25	27	M	4.96	EO	240	I	3	None	None
26	31	F	0.62	EO	24	I	1	None	None
27	36	F	0.13	EO	2	I	4	Thymoma	None
28	54	F	4.8	LO	72	IIB	2	None	PB
29	35	F	0.96	EO	6	I	2	N.A.	None
30	50	F	1.05	EO	96	I	9	Thymic hyperplasia	PB
31	48	M	1.96	EO	36	IIB	2	None	PB
32	14	F	2.63	EO	2	I	1	None	None
33	21	M	3.01	EO	210	I	2	Thymic hyperplasia	PB
34	67	F	1.3	LO	24	I	2	None	PB
35	65	F	5.74	LO	50	IIA	6	None	PB
36	55	F	2.92	LO	14	IIB	4	None	PB
37	25	F	2.25	EO	24	I	2	None	PB
38	46	F	3.39	EO	8	I	2	N.A.	None
39	24	M	4.64	EO	1	I	1	None	None
40	70	M	3.01	LO	1	IIA	4	None	None
41	14	F	7.88	EO	0.1	I	3	None	None

*M, male; F, female; EO, early onset; LO, late onset; N.A., not available; PB, pyridostigmine bromide*.

**Table 2:** Characteristics of the study participants.

**Characteristics**	**Value (percentages)**	***p*** **-value**
**Gender**		
Male	17 (41.5%)	
Female	24 (58.5%)	
**MG type according to age at onset**		
EOMG	28 (68.3%)	
LOMG	13 (31.7%)	
**MG type according to clinical manifestations**		
OMG	23 (56.1%)	
GMG	18 (43.9%)	
**Anti-AChR antibody**		
Seropositive	34 (82.9%)	
Seronegative	7 (17.1%)	
Thymic abnormality^*^		
Normal	26 (72.2%)	
Thymic hyperplasia	6 (16.7%)	
Thymoma	4 (11.1%)	

* *The frequencies of thymic abnormalities from 36 MG patients which are collected in Table 1*.

*These did not include all the specimens used in the thymus experiment (Alteration of CD19 + IL-10 + B Cells in the Thymus of MG Patients)*.

### Human Cell Isolation

Fresh PBMCs were used after extraction for B cell isolation and all the analyses. PBMCs were isolated by lymphocyte separation medium (DAKEWE, Shenzhen, China) gradient centrifugation. B cell subsets were isolated by FACS Aria (Becton Dickinson, USA) based on their expression of CD19, CD24, and CD38 (Biolegend, San Diego, USA). The cell viability of B cell subsets was about 95% before sorting, and it turned to about 60% after sorting, using a TC20 automated cell counter (Bio-Rad Laboratories) and Trypan blue (Stemcell Technologies, Vancouver, Canada). CD19^+^ B cells and CD4^+^ T cells were also isolated by magnetic-bead purification with MACS kits (StemCell Vancouver, Canada).

For the preparation of thymic lymphocytes, fresh thymectomy tissue was immediately minced with scissors in PBS medium. Single-cell suspensions were obtained and washed by centrifugation at 4°C and 300 g for 5 min. Then, the thymic single cells were resuspended in RPMI 1640 (Gibco, Waltham, USA). Thymic lymphocytes were isolated by lymphocyte separation medium (DAKEWE, Shenzhen, China) gradient centrifugation.

### Cell Culture

PBMCs from MG patients and healthy controls were cultured in RPMI 1640 (Gibco, Waltham, USA) supplemented with 10% FCS (Gibco, Waltham, USA) in 24-well plates (1.0–1.5 × 10^6^ cells/500 μl per well). Recombinant soluble CD40L (1 μg/ml) (PeproTech, Cranbury USA), LPS (10 μg/ml) (Sigma-Aldrich, St. Louis, USA), and CpG (OND2006) (3.85 μg/ml, 500 nM) (InvivoGen, San Diego, USA) were added to the medium. For Breg cell incubation, PMA (50 ng/ml), ionomycin (1 μg/ml), and brefeldin A solution (5 μg/ml) were added to the culture medium for 5 h.CD24^hi^CD38^hi^-depleted CD19^+^ B cells, and the overall population of CD19^+^ B cells (undepleted B cells) were added to 96-well plates with 0.5 μg/ml purified plate-bound CD3 mAb (Biolegend, San Diego, USA). T cells by magnetic-bead purification were also added at a ratio of 1:1 for coculture on plate-bound CD3 mAb for 72 h.

### Cytokine Detection

For analysis of human intracellular cytokine production, PBMCs were stimulated with PMA (50 ng/ml), ionomycin (1 μg/ml), and brefeldin A solution (5 μg/ml) for 5 h. T cells and B cells were stimulated with 0.5 mg/ml purified plate-bound CD3 mAb for 3 days. For intracellular staining, cells were stained with combinations of FITC antihuman CD4 (5 μl/test), PerCP/Cy5.5 antihuman CD19 mAb (5 μl/test), PE antihuman CD24, and FITC antihuman CD38 (Biolegend, San Diego, USA). Cells were washed, fixed, permeabilized (Cytofix/Cytoperm, BD, USA), and stained for the detection of intracellular cytokines with PerCP/Cy5.5 antihuman IFN-γ (5 μl/test), PE antihuman TNF-α (5 μl/test), and APC antihuman IL-10 mAb (5 μl/test) (Biolegend, San Diego, USA). Appropriate isotype controls were used for gate setting for cytokine expression.

### RNA Isolation and Quantitative PCR

Messenger RNA was isolated using the MiniBEST Universal RNA Extraction Kit (TaKaRa). cDNA was transcribed using PrimeScript RT Master Mix (TaKaRa) according to the manufacturer's protocol. Real-time PCR was performed using SYBR Premix Ex Tap II (TaKaRa). The *IL-10* primer sequences were 5′-ATAACTGCACCCACTTCCCA-3′ and 5′-TCATTTCCGATAAGGCTTGG-3′. The PCR conditions were as follows: 30 s at 95°C, followed by 40 cycles for 5 s at 95°C, 30 s at 60°C, and 15 s at 95°C.

### Cell Signaling

Cells were then stimulated by incubation with 5 mg/ml purified stimulatory mouse -antihuman CD40 mAb (5 μg/ml) (clone: 5C3, Biolegend, San Diego, USA) for 30 min on ice in the dark. Cells were washed, fixed, and permeabilized (Phosflow™ Fix buffer/Perm Buffer III, BD, USA) according to the manufacturer's instructions. Cells were washed with ice-cold PBS, resuspended in warm PBS at a density of 0.5–1 × 10^6^ cells/100 μl, incubated with Alexa Fluor® 647 mouse anti-STAT3 antibody (20 μl/test) (clone: pY705) (BD, USA) for up to 60 min at RT, washed and resuspended in Permwash buffer (BD, USA).

### Histology

Thymi of MG patients and controls were collected and fixed in 4% paraformaldehyde (pH 7.4) for 8 h. After dehydration in 30% sucrose, the thymi were embedded in Tissue OCT Medium (Sakura, Torrance, USA) and cut into 10-μm sections with air drying. To evaluate routine histopathological findings, some sections were stained with hematoxylin–eosin and examined by light microscopy according to standard protocols. For immunofluorescence, other sections were blocked with 3% bovine serum albumin (BSA) for 30 min at room temperature and then incubated with mouse antihuman CD19 MAb (1:100, MAB1794, Millipore), rat antihuman IL-10 MAb (1:100, sc-53705, Sant Cruz) in 1% BSA at 4°C overnight and then incubated with the corresponding fluorochrome-conjugated secondary antibodies, namely, CY3 goat antimouse IgG (1:200, Abcam) and FITC goat antirat IgG (1:200, Abcam), in the dark for 50 min at room temperature, followed by DAPI (Invitrogen) costaining. Immunofluorescence control was directly incubated with fluorochrome-conjugated secondary antibodies. After a final wash step and mounting with fluorescent antifade mounting medium (HelixGen), the slides were examined under an Olympus FV-1000 confocal microscope.

### Statistics

All values are expressed as the mean ± SEM. Depending on the normal distribution of the data, we performed analysis by Student's *t*-test analysis and two-way ANOVA. We conducted a correlation analysis using the Pearson's correlation. All data were analyzed using the SPSS19.0 software (SPSS Inc., Chicago, USA).

## Results

### Breg Cells Decreased in the Peripheral Blood of MG Patients Compared With Healthy Controls

**Figure 1:**
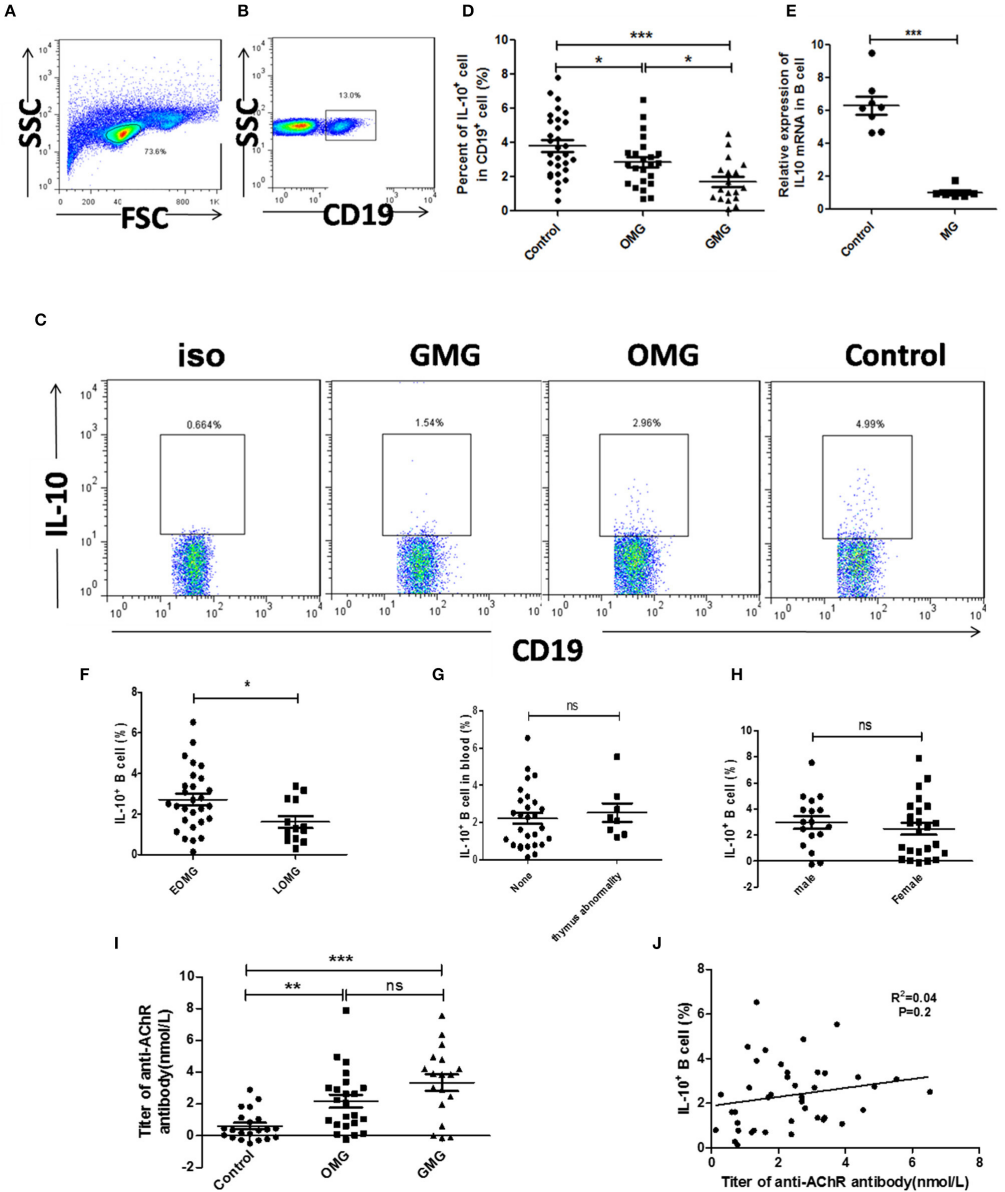
The proportion of CD19^+^ IL-10^+^ cells in peripheral blood cells of MG patients and controls. **(A)** Lymphocytes were gated according to forward scatter and side scatter by FCM. **(B)** B cells were gated according to CD19^+^ in lymphocytes. **(D)** Scatter plots show the mean percentages of CD19^+^ IL-10^+^ cells in the peripheral blood of 23 OMG patients, 18 GMG patients, and 30 healthy individuals. **(E)** Expression of IL-10 mRNA by real-time quantitative analysis in the peripheral blood of 8 MG patients and 8 healthy individuals. Six of 8 MG patients were ocular myasthenia gravis. **(C)** Representative flow cytometry plot of CD19^+^ IL-10^+^ cell gating for patients with OMG, GMG, and healthy controls. **(F)** The frequency of CD19+ IL-10+ cell in different MG types according to age at onset. **(G)** The frequency of CD19^+^ IL-10^+^ cells in different thymus status. **(H)** The frequency of CD19^+^ IL-10^+^ cells in different gender. **(I)** The titer of anti-AChR antibody in MG patients, including 21 healthy controls, 23 OMG patients, and 18 GMG patients. **(J)** The proportion of CD19^+^ IL-10^+^ cells had no correlation with the level of anti-AChR antibody, and the samples were from 41 patients with MG. *p*-values were calculated by Student's *t*-test; *p < 0.05, **p < 0.01, ***p < 0.001, ns, not significant.

First, we evaluated the proportion of total B cells and CD19^+^IL-10^+^ B cells by flow cytometry. Although there was no significant difference in total B cell count between MG patients and healthy controls (Supplementary Figure S1B), CD19^+^IL-10^+^ B cells were significantly decreased in the peripheral blood of MG patients compared with healthy controls. We further divided MG patients into OMG and GMG according to clinical manifestations. The frequency of CD19^+^IL-10^+^ B cells in the peripheral blood of GMG patients was much lower than that in the peripheral blood of OMG patients (*p* < 0.05; Figure 1C). Expression of IL-10 mRNA in B cells of MG patients (*n* = 8) was significantly lower than that in B cells of healthy controls (*n* = 8; *p* < 0.001; Figure 1D). Besides, the proportion of CD19^+^IL-10^+^ B cells in EOMG was higher than that of LOMG (*p* < 0.05; Figure 1F). However, there was no significant difference in CD19^+^IL-10^+^ B cells between different gender and different thymus status (Figures 1G, H).

We also detected the titers of anti-AChR antibody in sera of MG patients by ELISA. The level of anti-AChR antibodies in sera MG patients was significantly higher than that in sera of healthy controls (*p* < 0.001). There was no difference in the level of anti-AChR antibody between OMG and GMG (Figure 1I). Furthermore, there was no correlation between CD19^+^IL-10^+^ B cells and the titers of anti-AChR antibody in sera of MG patients (*R*^2^= 0.04, *p* = 0.2) (Figure 1J).

**Figure 2:**
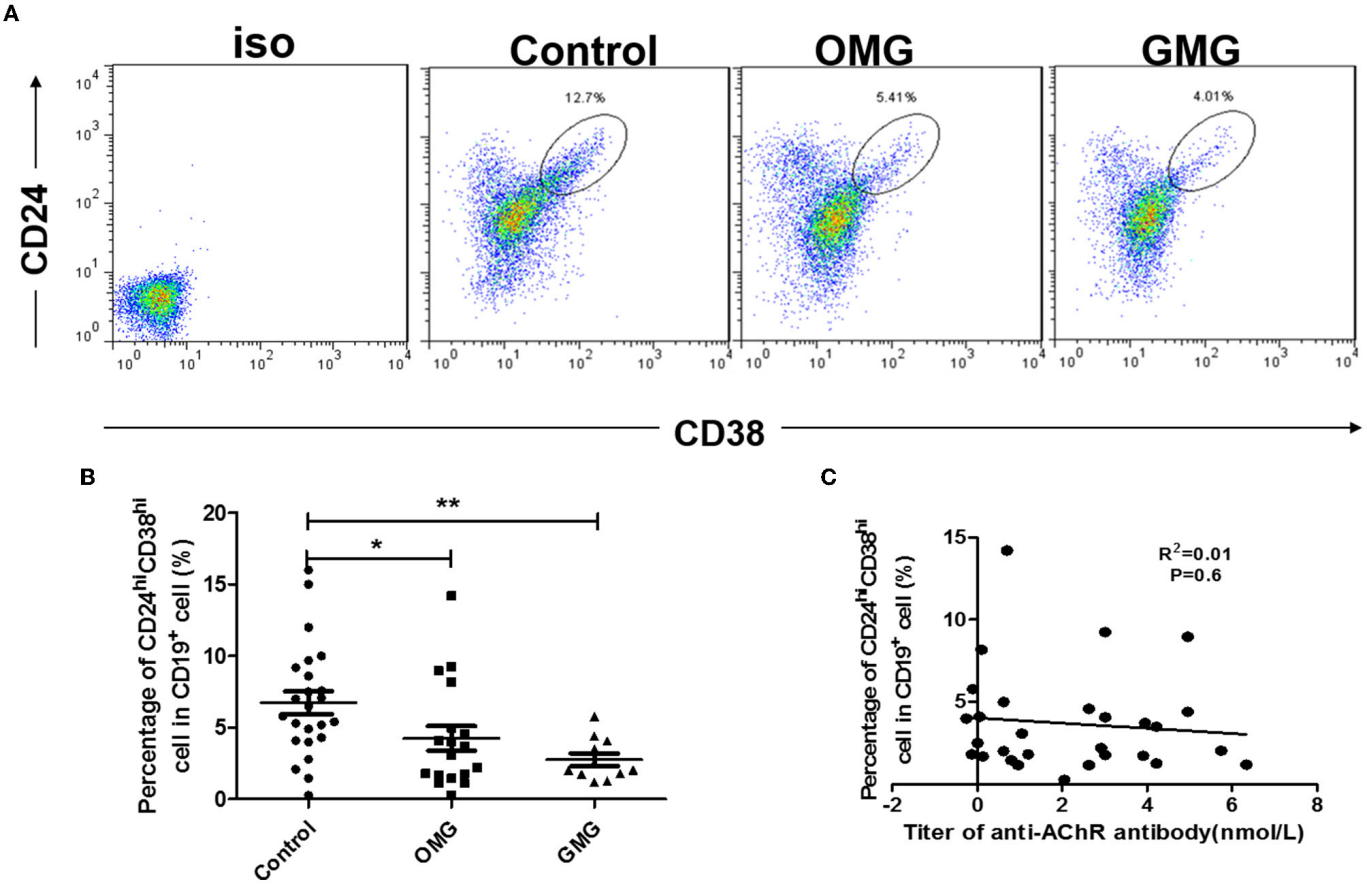
The proportion of CD19^+^CD24^hi^CD38^hi^ B cells in peripheral blood cells of MG patients and controls. **(A)** Representative flow cytometry plot of CD19^+^CD24^hi^CD38^hi^ B cell subset gating for patients with OMG, GMG, and healthy controls. **(B)** Scatter plots show the mean percentages of CD19^+^CD24^hi^CD38^hi^ B cells within each subset in the peripheral blood of 18 OMG patients, 11 GMG patients, and 24 healthy individuals. **(C)** The proportion of CD19^+^CD24^hi^CD38^hi^ B cell was uncorrelated with the level of AChR antibody, and the samples were from 41 patients with MG. *p*-values were calculated by Student's *t*-test; *p < 0.05, **p < 0.01.

We determined that CD19^+^CD24^hi^CD38^hi^ B cell subset was the main subset secreting IL-10 cytokines (Supplementary Figure S2). The proportion of CD19^+^CD24^hi^CD38^hi^ B cell subset in MG patients was also significantly lower than that in matched healthy controls (*p* < 0.05), whereas there was no significant difference in the proportion of CD19^+^CD24^hi^CD38^hi^ B cell subsets between the GMG and OMG patients (Figures 2A, B). However, the proportion of CD19^+^CD24^hi^CD38^hi^ B cell was uncorrelated with the level of AChR antibody (Figure 2C).

### Alteration of CD19^+^IL-10^+^ B Cells in the Thymus of MG Patients

**Figure 3:**
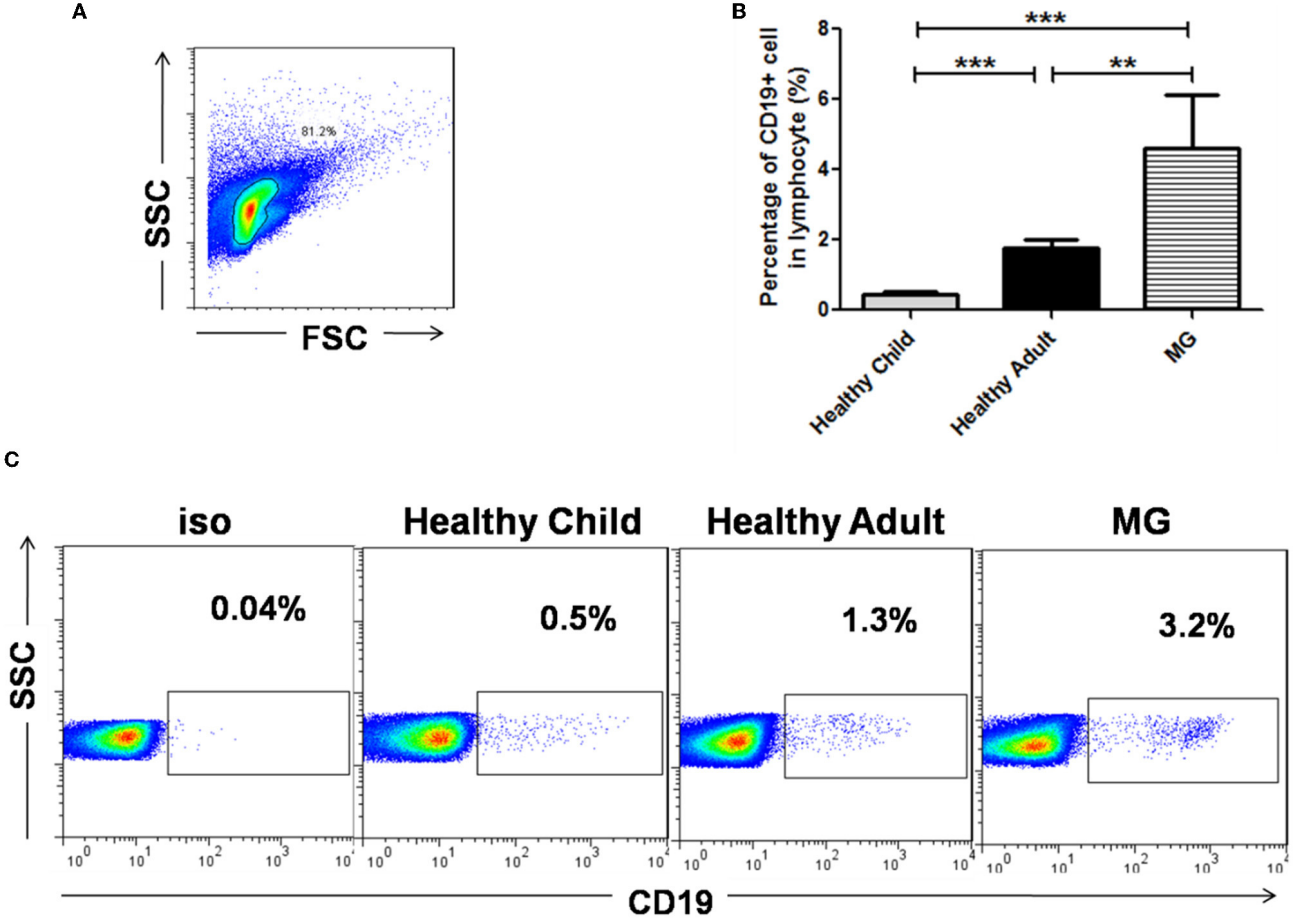
The proportion of CD19^+^ B cells in the thymus of MG patients and healthy individuals. **(A)** Lymphocytes were gated according to forward scatter and side scatter by FCM in the thymus of a healthy individual. **(B)** Bar charts showing the mean percentage ± SE of B cells in the thymus of 10 healthy children, 4 healthy adults, and 13 MG patients; **p < 0.01, ***p < 0.001. **(C)** CD19+ B cells in the thymus of a healthy child, a healthy adult, and an MG patient were gated by FCM.

We compared the total number of B cells in the thymus of healthy children (*n* = 7), healthy adults (*n* = 3), and MG patients (*n* = 4). The ratio of CD19^+^ B cells to thymocytes in MG patients (~4.5%) was significantly higher than that in healthy children (*p* < 0.001) and healthy adults (*p* < 0.01). The proportion of CD19^+^ B cells to thymic lymphocytes was ~0.55% in healthy children and ~1.8% in healthy adults. However, the frequency of B cells in healthy adults was significantly higher than that in healthy children (*p* < 0.001; Figure 3). It was found that with increasing age, the frequency of B cells in the thymus increased, and in the state of MG disease, the frequency of B cells was significantly higher than that of normal cells.

**Figure 4:**
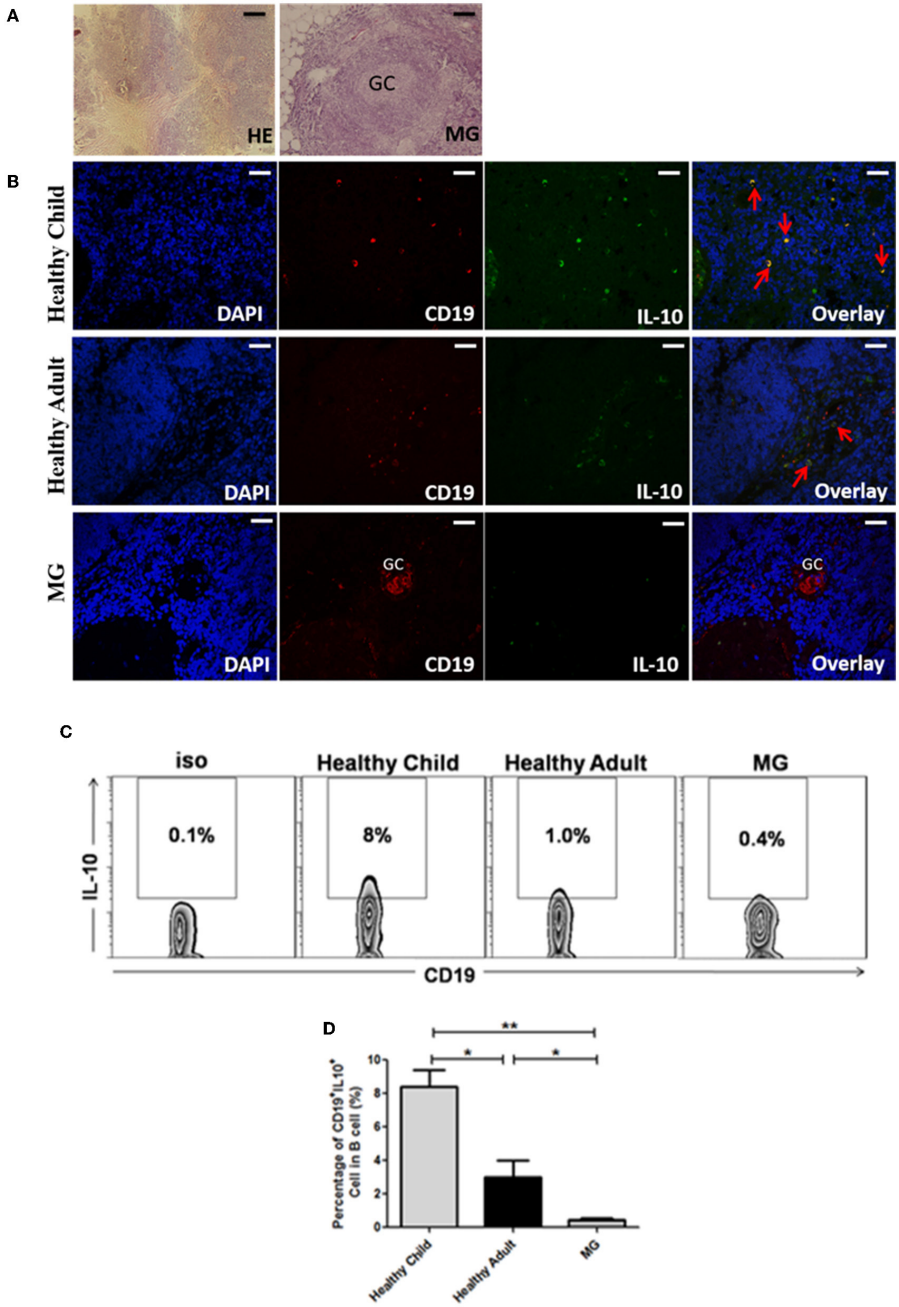
CD19^+^IL-10^+^ Breg cells were assessed in the thymus of healthy individuals and MG patients by immunofluorescence staining. **(A)** Human normal thymus and hyperplastic thymus from MG patients stained with hematoxylin and eosin. In addition, thymic hyperplasia was observed in an MG patient, which showed a germinal center (GC) structure (scale bars, 100 μm). **(B)** Frozen sections of thymic tissues were immunoassayed with nuclear DAPI staining (blue). Double immunofluorescence analysis was performed with anti-CD19 (red) and anti-IL-10 (green) antibodies. Note that CD19^+^IL-10^+^ Breg cells both express CD19 and IL-10 (yellow, with red arrow). It showed a germinal center (GC) structure in the hyperplastic thymus from MG patient (scale bars, 200 μm). Immunofluorescence control showed negative (not shown). **(C)** Representative dot plot showing the percentage of CD19^+^IL-10^+^ Breg cells in CD19^+^ B cells in the thymus of healthy children, healthy adults, and MG patients. **(D)** Bar charts showing the mean percentage ± SE of CD19^+^IL-10^+^ Breg cells in CD19^+^ B cells in the thymuses of healthy children (*n* = 10), healthy adults (*n* = 4), and MG patients (*n* = 13, 5 of MG thymuses are thymoma and 7 of MG thymuses are thymic hyperplasia); *p < 0.05, **p < 0.01.

Next, we studied the IL-10-secreting B cells (as Breg cells) expression in the thymus of healthy children, healthy adults, and MG patients by immunofluorescence staining. The thymus specimens of MG patients were all thymic hyperplasia. Result showed that Breg cells in the thymus of healthy children, healthy adults, and MG patients were mainly concentrated in the thymic medulla, cortical medulla, and perivascular tissues (Figures 4A, B). The frequencies of CD19^+^IL-10^+^ B cells in the thymus of healthy children (*n* = 7), healthy adults (*n* = 3), and MG patients (*n* = 4) were also compared by flow cytometry. It was found that the proportion of CD19^+^IL-10^+^ B cells in MG patients was significantly lower than healthy children (8 ± 1.9%) (*p* < 0.01) and healthy adults (3 ± 1.4%) (*p* < 0.05; Figures 4C, D).

### Impaired Function of Breg Cells in Inhibiting the Production of Inflammatory Cytokines by Th1 Cells in MG Patients

**Figure 5:**
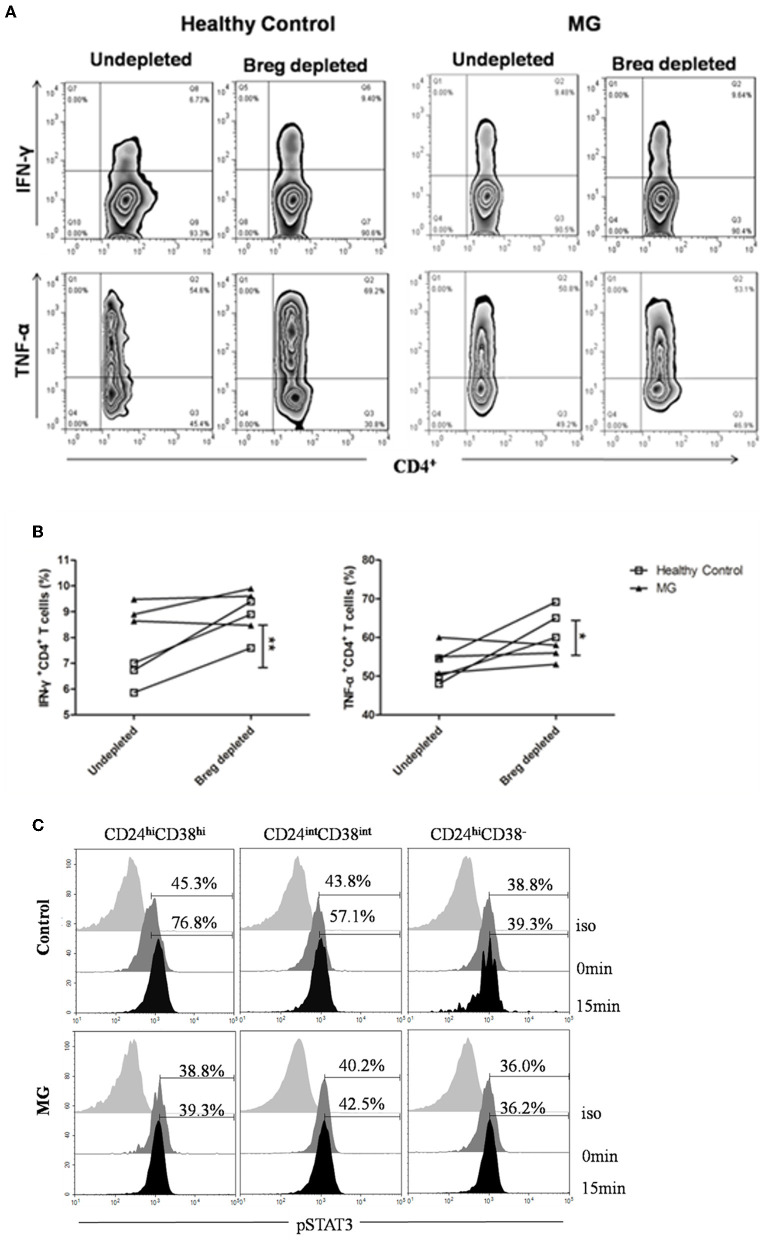
CD19^+^CD24^hi^CD38^hi^ B cells from MG patients fail to suppress IFN-^γ+^ and TNF-α^+^CD4^+^ T cell differentiation. B cells isolated from patients with MG or healthy controls were stained with CD19, CD24, and CD38 and gated, as shown in Figure 2A. CD19^+^CD38^hi^CD24^hi^ B cells were depleted, and the depleted B cells and undepleted B cells were both collected using flow cytometry sorting. CD4^+^ T cells were collected by magnetic-bead purification, and then, B cells and CD4^+^ T cells were cocultured for 72 h with 0.5 mg/ml plate-bound CD3 mAb. PMA+iono was added for the last 6 h of culture. Depleted and undepleted B cells were surface stained with CD4 and CD19 mAbs, permeabilized, and stained with TNF-α or IFN-γ mAbs. **(A)** Representative flow cytometry plots for IFN-γ^+^ and TNF-α^+^CD4^+^ T cells in MG patients and healthy controls according to whether CD19^+^CD38^hi^CD24^hi^ B cells were depleted or undepleted. **(B)** Graphs showing the differences in the frequency of IFN-γ^+^CD4^+^ and TNF-α^+^CD4^+^ T cells between depleted and undepleted B cells from the same individual. Healthy individuals are indicated by white squares, and MGs are indicated by black triangles. The results from three healthy individuals and three MG patients are shown. *p < 0.05, **p < 0.01. **(C)** Representative histograms of pSTAT-3 expression by CD19^+^CD24^hi^CD38^hi^, CD19^+^CD24^int^CD38^int^, and CD19^+^CD24^+^CD38^−^ B cells at 0 and 15 min post-stimulation with CD40 mAb for one MG patient and one healthy control.

Because of the low frequency of CD19^+^CD24^hi^CD38^hi^ cells, it is difficult to obtain sufficient CD19^+^CD24^hi^CD38^hi^ B cell numbers from peripheral blood samples, so we evaluated whether depletion of CD19^+^CD24^hi^CD38^hi^ B cells might impact T cell cytokine production. B cells (CD19^+^CD24^hi^CD38^hi^ cells depleted or undepleted) were separated from MG patients and healthy donors by flow cytometry sorting. Undepleted and depleted B cells were cocultured with autologous CD4^+^ T cells, respectively. We observed a significant increase in the frequency of CD4^+^IFN-γ^+^ T cells and CD4^+^TNF-α^+^ T cells in the CD19^+^CD24^hi^CD38^hi^ B cell-depleted group compared with the undepleted group from healthy donors. In contrast to healthy donors, the group depletion of CD19^+^CD24^hi^CD38^hi^ B cells in MG patients did not lead to any significant increase in the percentages of CD4^+^IFN-γ^+^ T cells or CD4^+^TNF-α^+^ T cells (Figures 5A, B).

CD40 ligation induced the expression of the IL-10 cytokine in B cells from human peripheral blood samples. Among CD40-triggered signals, the STAT3 (signal transducer and activators of transcription family) cascade has been measured with flow cytometry in peripheral blood samples (*n* = 2) (15). *In vitro* stimulation of peripheral blood cells derived from healthy donors (*n* = 2) with an agonistic CD40 mAb led to significantly higher p-STAT3 expression in CD24^hi^CD38^hi^ B cells than in either CD24^int^CD38^int^ B cells or CD24^hi^CD38^−^ B cells. However, there was no significant difference in the expression levels of p-STAT3 among the other two subsets of B cells in patients with MG (Figure 5C).

## Discussion

In this study, we basically studied the Breg cells alteration in the peripheral blood and thymus of MG patients. Through the paired test, we showed that not only the percentage of Breg cells decreased in MG patients, but also the function of Breg cells was impaired. CD40L-induced STAT3 signaling pathway may be involved in the loss of function in Breg cells of MG patients.

Breg cells are indispensable for the maintenance of tolerance and immune homeostasis, despite representing fewer than 10% (~4% in our study) of B cells in circulation in healthy individuals (16). Breg-mediated suppression occurs primarily *via* Il-10 production. Therefore, IL-10 is often recognized as a marker for Breg identification. Tedder et al. defined the group of B cells secreting IL-10 as B10 cells for the first time and considered that the B cells of CD1d^hi^CD5^+^ were the B cells that mainly secreted IL-10 in mice. However, Blair et al. have pointed out that CD19^+^CD24^hi^CD38^hi^ cells are the main B cell subsets secreting IL-10 in human peripheral blood (16). In addition, CD19^+^CD24^hi^CD38^hi^ B cells have been mostly demonstrated as Breg cells in humans (16–19). Therefore, our study also confirms that CD19^+^CD24^hi^CD38^hi^ cells in human peripheral blood were the B cell subsets that mainly secrete IL-10.

Although Bregs are indispensable for the maintenance of tolerance and immune homeostasis, its alteration in autoimmune diseases was diverse. Iwata et al. first reported that human IL-10-producing B lineage cells (IL-10^+^ B cells) were increased in various autoimmune diseases, such as SLE, RA, MS (multiple sclerosis), Sjögren's syndrome, and blistering skin diseases (20). In contrast, the proportions of Breg cells in other autoimmune diseases, such as MS (21), psoriasis (22), RA (23), and antibody-associated vasculitis (24), were significantly lower than that in healthy controls. In our study, the proportions of CD19^+^ IL-10^+^ cells and CD19^+^CD24^hi^CD38^hi^ cell subsets in the peripheral blood of MG patients were significantly decreased compared with those of healthy controls, which indicated that Breg cells were involved in the pathogenesis of MG. Moreover, there were functional deficits of Breg (CD19^+^CD24^hi^CD38^hi^) cells on Th1 cells to produce inflammatory cytokines. Jianrong Sheng's studies showed that CD1d^hi^CD5^+^ Breg cells could prevent the development and block the progression of disease in experimental autoimmune myasthenia gravis (EAMG) (25), and the immunomodulatory function of CD1d^hi^CD5^+^ Breg cells depended on the expression of IL-10 (26). In addition, the frequency of Breg cells was decreased in MG patients and inversely correlated with disease severity (27, 28).

MG is the only autoimmune disease closely related to the thymus, with 80–95% of MG patients experiencing thymic abnormalities and 20–25% of the patients having thymoma. Although a large number of previous studies have focused on the abnormality of T cells in the MG thymus, more and more studies have revealed the important role of B cells in it. Additionally, ectopic germinal centers have been detected by immunofluorescence staining of thymus in MG patients (29). The proportion of CD19^+^B cells in thymocytes in MG patients was significantly higher than that in healthy children and healthy adults, which was consistent with our past research (29). A previous study reported that B cells account for 0.1–0.5% of thymocytes in humans (30). In our present experiment, CD19^+^ B cells accounted for about 0.23% thymocytes of healthy children and about 0.54% thymocytes of healthy adults. To observe the ratio of B cells to thymic lymphocytes, we used lymphocyte separation solution to further purify the lymphocytes in thymocytes. The proportion of purified CD19+ B cells to thymus lymphocytes was ~0.55% (healthy children) and ~1.8% (healthy adults). It can be seen that the frequency of B cells in the thymus increases with age and especially more in MG patients which was consistent with previous report about B cell infiltration, germinal center formation in hyperplastic MG thymus (31). Yilmaz et al. found a few CD19^+^IL10^+^ cells in medulla and also within germinal centers in the thymus of MG patient but no CD19^+^IL10^+^ cell in control thymuses (32). However, our study confirmed that Breg cells existed in healthy adults (*n* = 4) and healthy children (*n* = 10). The difference between our results may due to the frozen tissues we used in our study would maintain the integrity of the antigen. Most importantly, we compared the changes in Breg cells in the thymus between MG patients and normal controls for the first time by flow cytometry. The results of flow cytometry were similar to that of immunofluorescence.

Chen Xing et al. found a group of IL-10-secreting CD19^+^CD5^+^CD1d^high^ cells in the mouse thymus. In an *in vitro* culture environment, this group of B cells can regulate the differentiation of CD4^+^Foxp3^+^ Treg cells. This study confirmed that Breg cells do exist in the mouse thymus and that these cells play an immunoregulatory role. Lu et al. reported that CD19^+^IL-10^+^ B cells also exist in the human thymus and found that CD19^+^IL-10^+^ B cells in the MG thymus were increased by semiquantitative analysis of the fluorescent spot area (33). However, our results indicate the opposite. We found that the frequency of CD19^+^IL-10^+^ B cells in the thymus of MG patients was much lower than that of healthy controls by flow cytometry, although the total number of B cells in the thymus of MG was greater than that of healthy controls. The difference in results may be due to different methods and the different histological types we used. According to the frequency of Breg cells in peripheral blood in humans, we thought that Breg cells were deficient in the thymus of patients with MG. In addition, the total number of B cells in the thymus of children was less than that in healthy adults, but the proportion of Breg cells in B cells was the highest in children among the three groups. This suggests that Breg cells may also play an immunomodulatory role in the human thymus.

It should be noted that when we studied the number of Breg cells in thymus tissue collected from MG patients, the tissue appeared to exhibit signs of thymic hyperplasia. We should collect other pathological types of thymus tissues, such as that of thymoma, in the future. We investigated whether STAT3 phosphorylation (p-STAT3) was different in CD24^hi^CD38^hi^ B cells isolated from MG patients in response to CD40L and CpG. The results indicated that the defective response to CD40 signaling in CD24^hi^CD38^hi^ B cells may be limited to certain cellular functions, in particular STAT3 phosphorylation and IL-10 production. This observation requires further study for confirmation.

Myasthenia gravis is a prototypical autoimmune disease, and most patients with MG require induction therapy with high doses of corticosteroids and maintenance with an immunosuppressant. Although steroids and other antiinflammatory drugs are effective therapies, patients can experience side effects, such as renal toxicity and other adverse effects, while achieving long-term remission. An increasing number of biologic agents are targeted therapies that can be selected to maximize the possibility of remission based on the immunopathogenesis of the disease. We observed that abnormal proportions and functions of Breg cells cause deleterious effects and trigger autoimmunity in MG patients. A better understanding of human Breg and other immune cell interrelations, specifically in the thymus of MG patients, is worth pursuing so that new therapeutic strategies can be developed. Breg cell therapy for MG warrants further investigation.

## Data Availability Statement

The original contributions presented in the study are included in the article/Supplementary Material, further inquiries can be directed to the corresponding author/s.

## Ethics Statement

The studies involving human participants were reviewed and approved by Ethics Committee of FMMU. The patients/participants provided their written informed consent to participate in this study.

## Author Contributions

TC, KY, and ZL contributed to conception and design of the study. YL organized the database. RL performed the statistical analysis. JL wrote the first draft of the manuscript. CS, CW, and JZ wrote sections of the manuscript. All authors contributed to manuscript revision, read, and approved the submitted version.

## Funding

This work was supported by the National Natural Science Foundation of China (Grant No. 81671233) and the Discipline Innovation and Development Plan of Tangdu Hospital-Major Program of Clinical Research (Grant No. 2021LCYJ002).

## Conflict of Interest

The authors declare that the research was conducted in the absence of any commercial or financial relationships that could be construed as a potential conflict of interest.

## Publisher's Note

All claims expressed in this article are solely those of the authors and do not necessarily represent those of their affiliated organizations, or those of the publisher, the editors and the reviewers. Any product that may be evaluated in this article, or claim that may be made by its manufacturer, is not guaranteed or endorsed by the publisher.
