# Supplementary material for: Regulatory B Cells Are Decreased and Functionally Impaired in Myasthenia Gravis Patients
Source: Front Neurol. 2022 Feb 28;13:808322. doi: 10.3389/fneur.2022.808322 (PMC8918563; doi:10.3389/fneur.2022.808322)
Supplement: Supplementary file 1 [file Data_Sheet_1.docx]

Supplementary Material

## Supplementary Figures


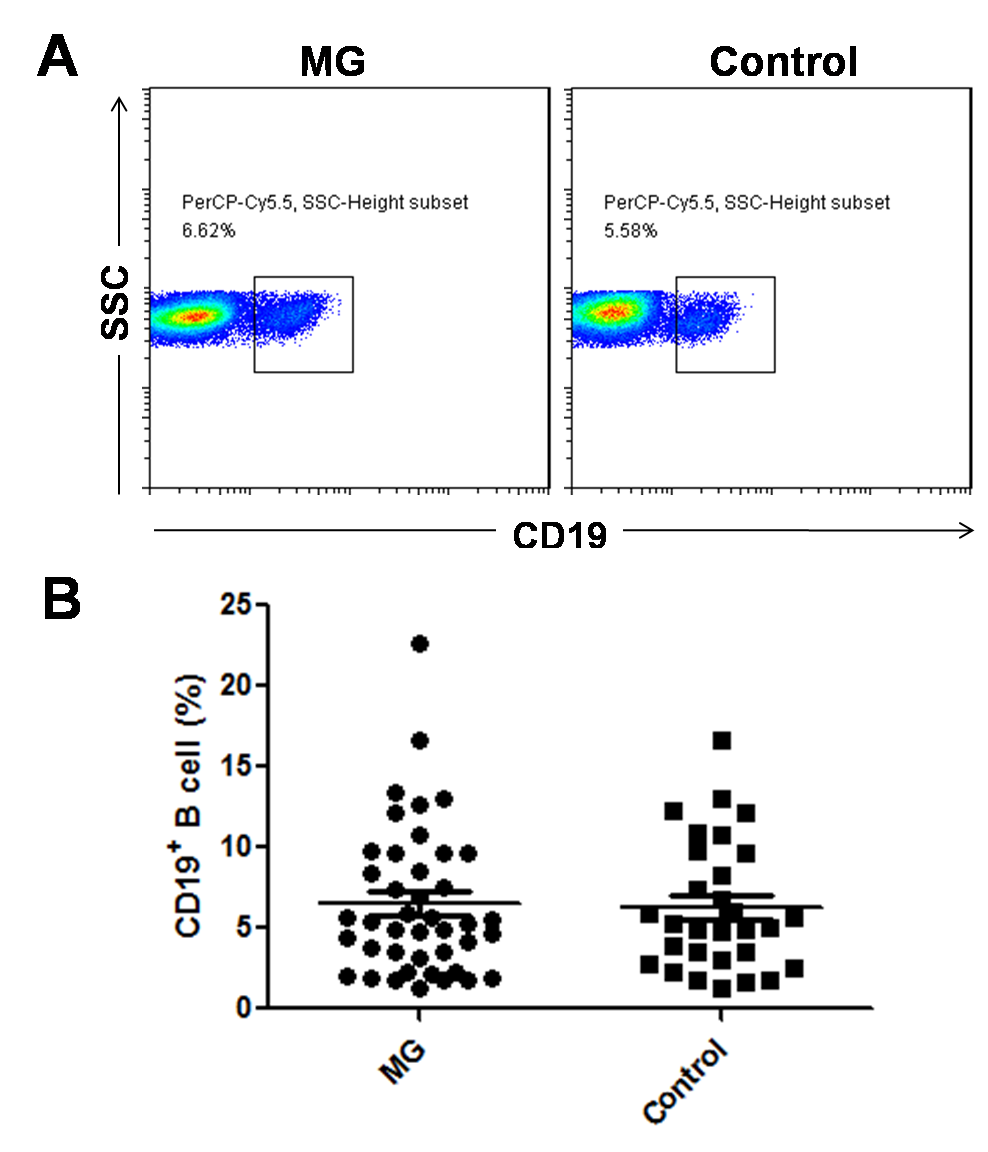


**Supplementary Figure 1.** **The proportion of CD19^+^ B cell in peripheral blood cell of MG patients and controls.**

(A). B cells were gated according to CD19^+^ in lymphocytes；

(B). Scatter plots show mean percentages of CD19^+^ B cell in the peripheral blood of MG patients (n=41) and healthy individuals (n=30).


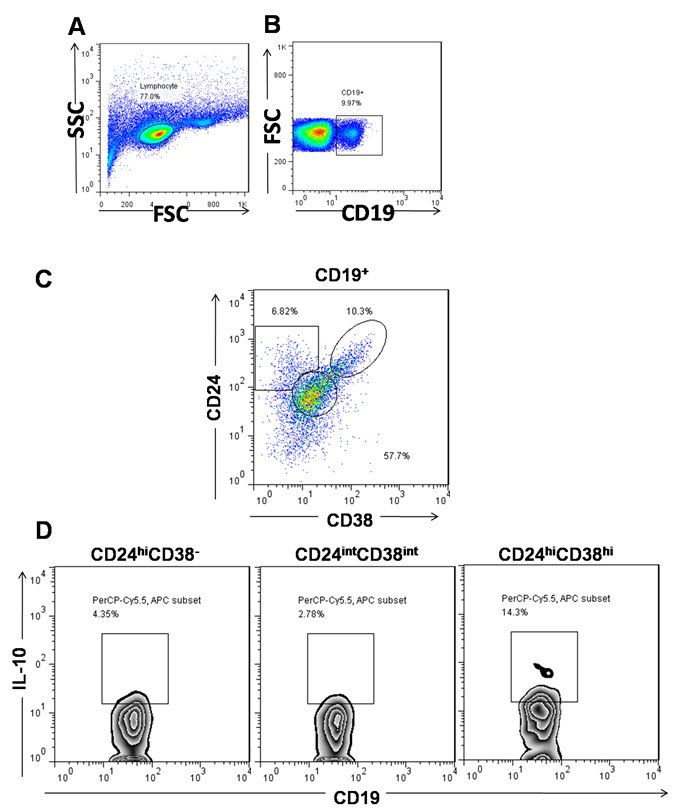
**Supplementary Figure 2. Identification of CD24 and CD38 as marker for Breg cells in human.**

(A) Lymphocytes were gated according to forward scatter and side scatter by FCM；

(B) B cells were gated according to CD19+ in lymphocytes；

(C) According to the marker of CD24 and CD38, B lymphocytes were divided into three groups: CD19^+^CD24^hi^CD38^hi^, CD19^+^CD24^hi^CD38^-^ and CD19^+^CD24^int^CD38^int^.

(D) Representative dot plot for one individual showing the frequency of IL-10 produced by different flow cytometry sorted B cell subsets.


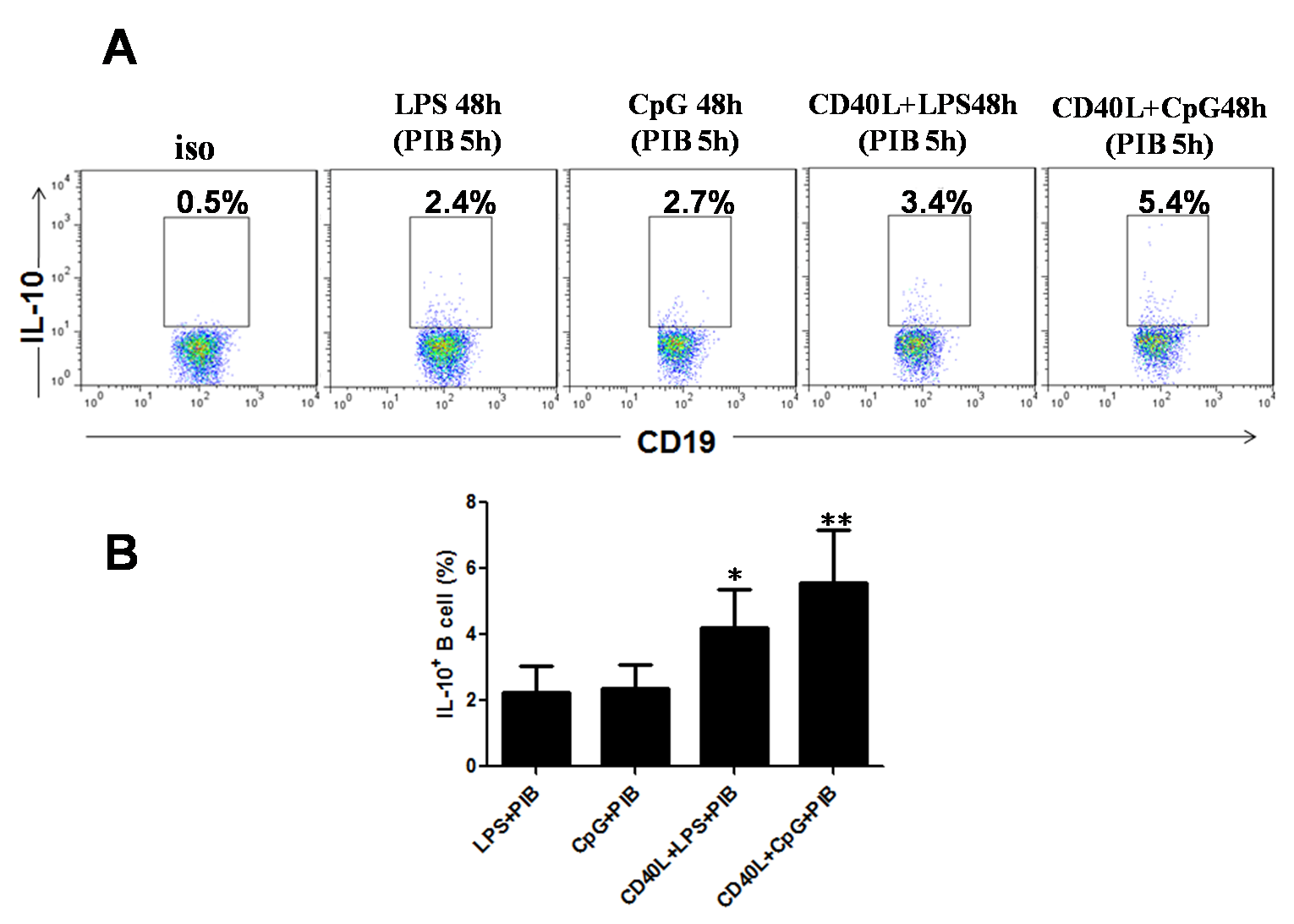


**Supplementary Figure 3. Proportion of CD19^+^ IL-10^+^ cells in different stimulation conditions.**

(A) CD19^+^ IL-10^+^ cells analyzed in different stimulation conditions by flow cytometry. iso: isotype control; PIB: PMA(Phorbol myristate acetate)+Inomycin+BFA(Brefeldin A solution);

(B) The percentage of IL-10^+^ B cells of total B cells in different stimulation conditions. Each group includes 3 healthy persons.
